# Comparative Diagnostic Accuracy of VistaCam IX Proxi and Bitewing Radiography for Detection of Interproximal Caries

**DOI:** 10.30476/dentjods.2022.95326.1860

**Published:** 2023-12-01

**Authors:** Solmaz Valizadeh, Yaser Safi, Azadeh Beigvand, Arash Farahnaki

**Affiliations:** 1 Dept. of Oral and Maxillofacial Radiology, Shahid Beheshti University of Medical Sciences, Tehran, Iran; 2 Undergraduate Student of Medicine, Kerman University of Medical Sciences, Kerman, Iran; 3 Postgraduate Student, Dept. of Orthodontics, Hamadan University of Medical Sciences, Hamadan, Iran

**Keywords:** Dental Caries, Diagnosis, Infrared Rays

## Abstract

**Statement of the Problem:**

Early detection of caries and the extent of carious lesions for appropriate treatment planning are very important and lead to introduction of new diagnostic tools.

**Purpose:**

This study aimed to compare the diagnostic accuracy of VistaCam IX Proxi and bitewing radiography for detection of posterior interproximal caries.

**Materials and Method:**

This *in vitro* study was performed on 40 extracted posterior teeth without cavitated carious lesions. Bitewing radiographs were obtained, infrared (IR) examination was performed, and the teeth were sectioned for histopathological analysis under a stereomicroscope as the gold standard for detection of caries and determination of the extent of carious lesions. Data were analyzed with Cohen’s kappa statistic, and Wilcoxon rank sum test.

**Results:**

The specificity of VistaCam IX Proxi and bitewing radiography was 71.4% and 87.7%, respectively. Their sensitivity was 100% and 40% for enamel caries, 72.8% and 54.5% for external half dentin caries, and 82.3% and 58.8% for internal half dentin caries, respectively (*p*= 0.048).

**Conclusion:**

Bitewing radiography had a higher specificity and lower rate of false positive results. However, VistaCam IX Proxi had higher sensitivity for caries detection with lower rate of false negative results. Considering the higher sensitivity and significantly lower frequency of false negative results by VistaCam IX Proxi, it may be reliably used for caries detection specially enamel caries, and can serve as an adjunct to bitewing radiography.

## Introduction

In spite of scientific advances and the improved status of public health, dental caries has remained a noticeable dilemma [ 1 ]. The development course of caries is both preventable and stoppable. In the case of early detection of caries, noninvasive methods including antimicrobial therapy, fluoride therapy, low-level laser therapy, and diet modification, may be used to stop or even reverse the caries process [ 2 ]. The most practical diagnostic tools used in clinical practices are radiological and clinical examination. Although both of these diagnostic tools feature high specificity, they manifest low sensitivity. As a result, some incipient caries may be missed. Given the impossibility of direct observation and contact with the adjacent teeth, the detection of inter-proximal caries is accompanied by many difficulties. Thus, interproximal carious lesions are hardly detectable in their initial stages [ 3 - 4 ]. 

In recent years, a number of techniques have been presented in order to improve the detectability of interproximal caries without the need for radiography, including laser fluorescence and fiber optic transillumination [ 3 - 4 ]. The use of infrared (IR) and near IR wavelengths is a new technology based on digital imaging fiber optic transillumination for caries detection. The main difference between these two techniques is that while visible light is used in digital imaging fiber optic transillumination, the other system employs invisible light characterized by a long wavelength [ 5 - 9 ]. Some of the new systems introduced to the market for this purpose are replaceable Proxi head for VistaCam IX intra-oral camera (Durr Dental, Bietigheim-bissingem, Germany) and Diagnocam (Kavo, Biberach, Germany) [ 3 , 10 ]. This device benefits from 2IR LED (850 nm wavelength) and the optical output part of this device measures 7×9mm. Following the radiation of light to the distal and mesial surfaces of the adjacent teeth, the radiation passes through the transparent enamel structure and is scattered by the carious lesion and enamel [ 11 - 12 ]. The CCD receptor receives the reflected and scattered lights. This phenomenon leads to the development of some white points on the image compared to healthy enamel. The image is then displayed by DBSWIN or VISTA SOFT programs [ 3 - 4 , 6 , 11 - 12 ]. Concerning the IR reflection and absorption spectrum, a number of scholars have recommended that waves ranging from 1300 to 1700 nm show the best potential of revealing caries in this technology. One can attribute this phenomenon to favorable absorption and low scattering within the above range, leading to the provision of superb contrast for differentiation between the sound enamel and carious lesions [ 13 - 14 ]. Nonetheless, when choosing the characteristics of the intra-oral devices for *in vivo* circumstances, one should consider the effect of water on/within the surface of enamel as the most important effective parameter. Thus, the appropriate wavelength used to capture the best diagnostic image is 850 nm [ 4 ]. Evidence suggests that one can use IR images in order to detect demineralization beneath sealants, buccal surface caries, and secondary caries beneath composite restorations and determine the extent/severity of occlusal caries and also the extent of water loss of the structure of teeth in the course of demineralization [ 10 , 15 - 22 ]. This system is supposed to serve as a superb diagnostic system, in particular for the follow-up of patients at high risk of caries, children, pregnant women, and patients suffering large torus, an extreme gag reflex, as well as the sites, which are hardly examinable through radiography [ 10 ]. Early detection of caries is highly important in order to prevent invasive treatments, especially in patients for whom radiography is contraindicated. This investigation aimed to study the accuracy of VistaCam IX Proxi with IR light at a wavelength of 850 nm compared to bitewing radiography for detecting interproximal caries.

## Materials and Method

**Figure 1:**
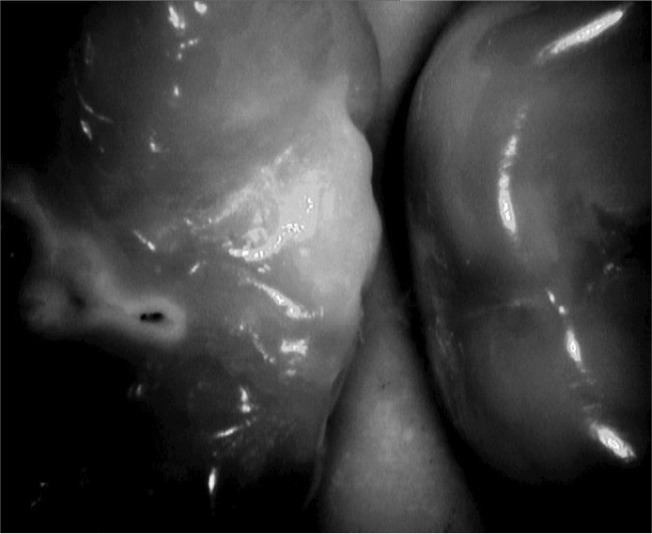
Vistacam IX Proxi’s real time image

The present *in vitro* diagnostic investigation was carried out on 40 extracted human permanent premolars and molars. The inclusion criterion of the study was sound teeth characterized by discoloration/non-cavitated incipient caries, which were unobservable directly when the teeth were in contact with each other. Teeth with cavitated lesions or restorations were excluded. By immersing in 1% sodium hypochlorite solution for 12 h, the collected teeth were disinfected, which were then stored in saline. Using two silicon blocks, which were in contact with one another, the selected teeth were mounted in such a way that the contact area between every two teeth simulated the clinical intraoral position of the teeth. Bitewing radiographs were obtained under similar conditions using a photostimulable phosphor plate receptor (ACTEON, France) and an intra-oral radiography unit (GENDEX, USA). Then, using VistaCam IX (Durr Dental, Bietigheim-bissingem, Germany), IR images were obtained from the proximal surfaces of the mounted teeth. Therefore, after drying the teeth, they were placed within a medium in a dimly lit room in order to simulate the oral cavity. Subsequently, the camera and also its special holder were adjusted over the occlusal surface at the contact area between the two teeth, and the image of interest was taken from the teeth in accordance with the instructions presented by the manufacturer (Figure 1). After image acquisition and briefing the observers regarding the correct observation of each series of images and the enhancement techniques, the images were evaluated by two oral and maxillofacial radiologists. Their opinions regarding the absence or presence of caries and the extent of interproximal lesions were recorded separately for each interproximal region using the criteria defined as (0) no caries at the contact area, (1) caries found in the enamel, (2) caries found in the outer half of dentin, and (3) caries found in the inner half of dentin.

**Figure 2:**
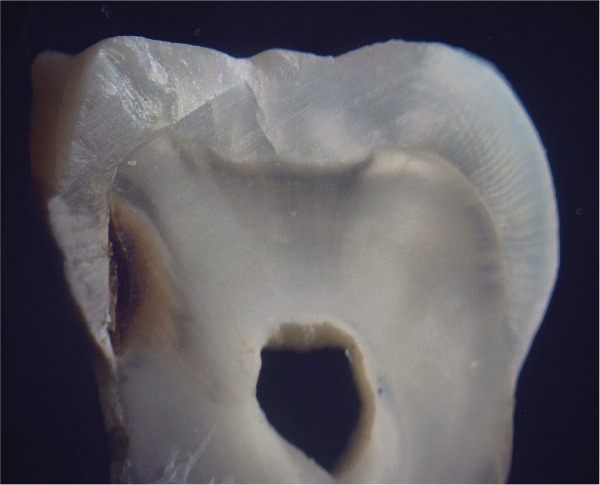
Histopathological analysis (gold standard)

After two weeks, the images were observed again by the same observers, and the intra- and inter-observer reliability values were calculated. Among the results, cases with a higher level of agreement between the two observations were set aside, and the differences were discussed by the two observers until a consensus was reached. After reaching a consensus, individual responses were recorded for the final comparison. SCAN-ORA software (Soredex, Tuusula, Finland) was used to observe and interpret the radiographic images, and DBSWIN software (Durr Dental, Bietigheim-bissingem, Germany)was utilized to observe and interpret the IR images. The teeth were then assessed by histological examination as the gold standard. For the same purpose, by employing a saw (Isomet; Buehler, USA), a minimum of three sections were prepared from each tooth at the site of its carious lesion. Then, the prepared sections were converted to microscopic slides observed via a stereomicroscope (Olympus, szx9, Japan) (see Figure 2). Absence or presence of caries and the extent of lesions were recorded by considering the defined criteria as (0) minimum caries, (1) caries found at the level of enamel, (2) caries found in the external half of dentin, (3) caries found in the internal half of dentin.

Next, a comparison was made between the results obtained by using each diagnostic tool and between the histological analysis results as the gold standard. As soon as data collection was completed, the data pertaining to the two observers were compared, and then the kappa coefficient of agreement between the two observers was estimated at two different time points. The relative and absolute frequency of the correct diagnoses obtained using each diagnostic tool were also reported. In addition, the kappa coefficient of agreement between the two techniques was estimated via the standard technique and reported. It is noteworthy that the sensitivity of each tool (bitewing radiography and VistaCam IX Proxi) was determined based on the extension of caries and reported. Eventually, the specificity value of each diagnostic tool for the detection of caries-free teeth was determined and reported. While the kappa statistic was used to analyze the agreements, the Wilcoxon rank sum test was utilized to make a comparison between the results obtained using the two diagnostic tools and the corresponding value of the gold standard. To analyze the obtained data, SPSS 21 was utilized. The Ethics Committee of Shahid Beheshti University of Medical Sciences (IR.SBMU.RIDS.REC.1395.365) verified the research protocol.

## Results

According to the results obtained through histological analyses as the gold standard, 7 out of 40 teeth evaluated in this study had absolutely no caries, while five teeth had enamel caries, 17 had caries in the internal half of dentin, and 11 had caries in the external half of dentin. The value of the intra-observer agreement for the first observer was 0.85, while it was 0.68 for the second observer when employing VistaCam. In addition, it was 0.69 for the second observer and 0.79 for the first observer in bitewing (see Table 1).

**Table 1:** Intra observer agreement

		VistaCam results				Total	bitewing results				Total
		Sound	Enamel caries	External half dentin caries	Internal half dentin caries		Sound	Enamel caries	External half dentin caries	Internal half dentin caries	
First observer	Sound	5	1	0	0	6	10	1	0	0	11
	Enamel caries	0	7	0	0	7	1	4	0	0	5
	External half dentin caries	0	0	9	3	12	0	0	10	2	12
	Internal half dentin caries	0	0	0	15	15	0	0	2	10	12
Total		5	8	9	18	40	11	5	12	12	40
Kappa agreement						0.85					0.79
Second observer	Sound	4	2	0	0	6	9	2	0	0	11
	Enamel caries	0	6	0	0	6	1	3	2	0	6
	External half dentin caries	0	0	8	5	13	0	0	8	1	9
	Internal half dentin caries	0	0	2	13	15	0	0	3	11	14
Total		4	8	10	18	40	10	5	13	12	40
Kappa agreement						0.68					0.69

The inter-observer agreement calculated for the first observation of VistaCam iX Proxi images was 0.82, while it was 0.56 for the second observation. The value of the inter-observer agreement for the results acquired from the first and the second bitewing assessments were 0.76 and 0.72, respectively (see Table 2).

**Table 2:** Inter observer agreement

		VistaCam results				Total	bitewing results				Total
		Sound	Enamel caries	External half dentin caries	Internal half dentin caries		Sound	Enamel caries	External half dentin caries	Internal half dentin caries	
Firstobservation	Sound	5	1	0	0	6	10	1	0	0	11
	Enamel caries	1	5	1	0	7	1	4	0	0	5
	External half dentin caries	0	0	11	1	12	0	1	8	3	12
	Internal half dentin caries	0	0	1	14	15	0	0	1	11	12
Total		6	6	13	15	40	11	6	9	14	40
Kappa agreement						0.82					0.76
Second observation	Sound	2	3	0	0	5	9	2	0	0	11
	Enamel caries	2	5	1	0	8	1	3	1	0	5
	External half dentin caries	0	0	6	3	9	0	0	10	2	12
	Internal half dentin caries	0	0	3	15	18	0	0	2	10	12
Total		4	8	10	18	40	10	5	13	12	40
Kappa agreement						0.56					0.72

Given the acquired agreement coefficients, in the first observation of both VistaCam IX Proxi images and bitewing radiographs, the first and second observers showed a higher agreement.

At last, the results acquired from the first observation were selected, and the disagreements between the two observers were resolved by consultation. As the final result was obtained from VistaCam IX Proxi and bitewing radiographs, a comparison was made between this result and the corresponding gold standard. To compare the final results with those obtained via the histological analysis as the gold standard, the kappa agreement coefficient and the Wilcoxon rank sum test were applied. The results obtained from comparing VistaCam IX Proxi and the gold standard are as follows (Table 3).

**Table 3:** Comparison the results of VistaCam ix proxy and DIAGNOdent with gold standard

		Gold standard				Total
		Sound	Enamel caries	External half dentin caries	Internal half dentin caries	
VistaCam	Sound	5	1	1	0	7
	Enamel caries	0	5	0	0	5
	External half dentin caries	1	1	8	1	11
	Internal half dentin caries	0	0	3	14	17
Total		6	7	12	15	40
Bitewing	Sound	6	0	0	1	7
	Enamel caries	2	2	1	0	5
	External half dentin caries	3	1	6	1	11
	Internal half dentin caries	0	2	5	10	17
Total		11	5	12	12	40

As Table 3 indicates, the value of the total agreement coefficient obtained for VistaCam was 0.718. Of a total of seven teeth without caries, VistaCam reported 1, 1, and 5 cases as cases with caries in the external half of dentin, cases with enamel caries, and sound, respectively. Therefore, its specificity for caries-free surfaces was 71.4%, and the reported percentage of false positive results was 28.5% approximately. In addition, among of dentin, one was reported as sound. As a result, the positive predictive value of VistaCam was calculated as 83.3%. All five samples featuring enamel caries were diagnosed correctly. Thus, the VistaCam sensitivity was 100% for the surfaces suffering enamel caries, while the reported percentage of false negative results was 0%. In addition, one sample with caries in the external half of dentin and one sound sample were reported among those enamel caries. Thus, the positive predictive percentage of the surfaces characterized by enamel caries was 71.4%. Among the eleven samples featuring caries in the external half of dentin, VistaCam correctly detected 8 cases with caries found in the external half of dentin, 1 with enamel caries, 1 without caries, and 1 with caries situated in the external half of dentin. Therefore, its sensitivity was 72.7% for the surfaces with caries in the external half of dentin, while the percentage of false negative results was 9%. Furthermore, one sample without caries and three samples with caries situated in the internal half of dentin were wrongly detected as those with caries situated in the external half of dentin by VistaCam. As a result, the positive predictive value of VistaCam reported for those surfaces that featured caries in the external half of dentin was 66.6%. Of 17 samples with caries situated in the internal half of dentin, the VistaCam correctly detected 14 cases; 3 cases were diagnosed with caries situated in the external half of dentin. As a result, the sensitivity of the device for surfaces characterized by caries in the internal half of dentin was 82.3%. On the other hand, it wrongly reported one sample with caries situated in the external half of dentin as having caries found in the internal half of dentin. Therefore, its positive predictive value for surfaces with caries situated in the internal half of dentin was 93.3%.

As Table 3 shows, the total agreement coefficient of bitewing radiography was equal to 0.449. Of the seven samples without caries detected on bitewing radiographs, six were correctly detected as sound and one had caries situated in the internal half of dentin. As a result, the estimated specificity of bitewing images for sound surfaces was 87.6%, while the percentage of false positive results was equal to 14.2%. In addition, two samples with enamel caries and three samples with caries in the external half of dentin were detected as sound on bitewing radiographs. As a result, the estimated negative predictive value of bitewing radiography was 54.5%. Two out of the five samples characterized by enamel caries on bitewing images were properly reported to have enamel caries, 1 was reported with caries in the external half of dentin, and 22 were reported as sound. As a result, the calculated sensitivity of bitewing radiography for enamel caries was equal to 40%; moreover, the percentage of false negative results was equal to 40%. In addition, bitewing radiography incorrectly reported one sample with caries found in the external half of dentin and also two samples with caries situated in the internal half of dentin as sound. As a result, the estimated positive predictive value of bitewing radiography for the surfaces featuring enamel caries was 40%. Of the 11 samples featuring caries in the external half of dentin, six were correctly diagnosed, one had enamel caries, three did not have caries, and one featured caries in the internal half of dentin. As a result, the calculated sensitivity of bitewing radiography for the surfaces featuring caries in the external half of dentin was 54.5% approximately, and the percentage of false negative results was equal to 27.2%. From another viewpoint, one sample featuring enamel caries and five samples featuring caries in the internal half of dentin were wrongly reported to have caries situated in the external half of dentin. Thus, the positive predictive value of bitewing radiography for the surfaces featuring caries in the external half of dentin was 50%. Of 17 samples with caries situated in the internal half of dentin, two, five, and ten samples were reported to have enamel caries, caries situated in the external half of dentin, and caries situated in the internal half of dentin (the latter being properly reported), respectively. As a result, the estimated sensitivity of bitewing radiography for the surfaces featuring caries in the internal half of dentin was 58.8%. Also, one sample without caries and one sample with caries in the external half of dentin were wrongly reported to have caries situated in the internal half of dentin. As a result, the estimated positive predictive value of bitewing radiography for the surfaces featuring caries in the internal half of dentin was 83.3%. The results of both VistaCam IX Proxi and bitewing radiography had significant differences compared to the gold standard (*p*= 0.048).

## Discussion

At present, with regard to the reversibility of caries if detected early before cavitation, and the reduction of caries prevalence in some parts of the world, finding more precise methods for caries detection has become increasingly important. Furthermore, various imaging methods are available to evaluate dental caries. Different studies in recent years have assessed the accuracy of various imaging systems and reported controversial results [ 15 - 22 ]. Based on studies, the different representation of demineralized tissue in comparison with other changes, such as developmental lesions, pigmentation, cracks, scales, and fluorosis, is an advantage of the IR images. Furthermore, since IR images are real-time, this characteristic enables the clinician to diagnose carious lesions, which could have remained undetected on bitewing radiographs. Nonetheless, the IR images also have a number of disadvantages, including inaccurate inspection of the depth of caries and distance from the pulp and incapability to examine the periodontal structure around the teeth [ 10 , 15 - 22 ]. Nevertheless, IR images have the ability to reveal proximal lesions [ 23 ]. The present investigation was carried out in order to compare and assess the diagnostic precision of VistaCam IX Proxi, which takes advantage of IR light in order to detect caries and bitewing radiography for the detection of proximal caries. The obtained results indicate that the estimated specificity of VistaCam IX Proxi for sound surfaces was equal to 71.4%, while it was 85.7% for bitewing radiography. This suggests that it is less likely that false-positive results occur in sound samples when using VistaCam compared to bitewing radiography, and it has a higher probability for correct detection of sound surfaces; thus, unnecessary treatments would be prevented. Previous studies have obtained contradictory results on this topic. For example, in an *in vitro* study by Maia Ama *et al*. (2011) comparing the IR radiation imaging and bitewing radiography techniques for the detection of incipient caries, it was observed that the specificity of images obtained by IR was higher than that of bitewing radiography [ 24 ]. However, in their *in vivo* investigation, Russotto *et al*. (2016) used IR images to detect proximal caries. They suggested that bitewing images were more specific than IR images [ 25 ]. Furthermore, Gokhan Ozkan *et al*. (2017) evaluated IR images for the detection of dentin proximal caries *in vivo*. Eventually, they found that the specificity of bitewing images was higher than that of IR images [ 3 ]. Schwendicke *et al*. [ 26 ] performed a systematic review and concluded that the specificity of bitewing radiography was higher than that of IR images. Considering enamel caries, since the determination of the extent of caries in the internal and external half of enamel would have no significant effect on preventive treatment planning, the extent of enamel lesions was not specified in this study, and such lesions were generally categorized as enamel caries. Considering enamel caries, while in VistaCam IX Proxi, the sensitivity was 100%, the sensitivity of bitewing radiography was 40%. This finding reflects the considerably higher sensitivity of VistaCam IX Proxi for the detection of incipient enamel carious lesions, which were reversible as well. Taking advantage of non-invasive approaches without the need for employing ionizing radiation will be favorable in preventive dentistry and the follow-up of high-risk patients. With regard to caries located in the external half of dentin, the sensitivity of bitewing radiography and VistaCam IX Proxi was 54.5% and 72.7%, respectively. This indicates the higher diagnostic accuracy and sensitivity of VistaCam in comparison with bitewing radiography for the follow-up of patients and detecting the extent of carious lesions. False negative responses in IR images are less likely to occur, while the probability of proper detection of caries situated in the external half of dentin is higher. With regard to caries situated in the internal half of dentin, the sensitivity of bitewing radiography and VistaCam IX Proxi was 58.8% and 82.3%, respectively, which indicates the higher sensitivity of the latter for more precise determination of the extent of caries. This finding suggests that the false negative results may hardly occur in IR images, while the probability of proper detection of caries in the internal half of dentin would be higher. Furthermore, investigation of the false detection of caries by bitewing radiography suggested that the majority of incorrect results were related to the cases where the extent of caries was underreported. As a justification, the minimum extent of demineralization of tooth structure required to be detectable on radiographs is around 35-40%. Therefore, the sensitivity of radiography is lower, and its results are typically underestimated. Furthermore, the comparison of the two modalities with the gold standard suggested a correlation coefficient of 0.449 for bitewing radiography and 0.718 for VistaCam. This reflects the higher capability of VistaCam IX Proxi in accurate diagnosis of the extent of caries. The results of previous studies on this topic are controversial. Maia Ama *et al*. (2011) made a comparison between the diagnostic accuracies of bitewing radiography and IR images for the detection of initial interproximal caries. They found that the IR images showed a higher sensitivity for caries diagnosis compared to bitewing radiography [ 24 ]. Russotto *et al*. evaluated IR images for the diagnosis of interproximal caries *in vivo*. They found that the IR images resulted in much more sensitivity for the diagnosis of interproximal caries. In addition, the occurrence of false positive responses was more probable, which approved the results of this investigation [ 25 ]. Nonetheless, Kuhnisch *et al*. (2016) evaluated the validity of IR radiation for the diagnosis of dentin interproximal caries. They showed that IR images and bitewing radiography had the same diagnostic accuracy for caries extended to dentin [ 9 ]. In their *in vivo* investigation of the diagnostic accuracy of IR radiation for caries diagnosis, Sochtig *et al*. (2014) observed that both techniques had the same detection accuracy for the diagnosis of occlusal and proximal caries [ 27 ]. Hakki Baltacioglu *et al*. (2017) made a comparison between these two diagnostic modalities for the detection of interproximal caries. They found no significant difference between the images obtained by the two techniques and suggested that IR radiation could be used as a suitable method with acceptable accuracy for caries detection [ 5 ]. Also, in their *in vitro* investigation, Abogazalah *et al*. (2017) compared these two techniques for the diagnosis of non-cavitated proximal caries. They found out that both techniques were of the same diagnostic accuracy for the diagnosis of non-cavitated proximal caries [ 28 ]. Jablonski-Momeni *et al*. (2017) evaluated VistaCam IX Proxi for the diagnosis of enamel caries. They observed no meaningful differences between the diagnostic accuracies of IR images and bitewing radiography for the diagnosis of enamel proximal caries [ 4 ]. Variations in the results of earlier investigations and the current findings are attributable to different conditions of the samples, such as performing the study only on initial enamel or dentin caries, different experimental conditions (*in vivo*/ *in vitro*), use of conventional or digital radiography with different sensors, different wavelengths of IR radiation, and employing other methods as the gold standard. Based on the obtained results and its availability, VistaCam IX Proxi seems to be an appropriate modality for the detection of caries with/without radiography. It is capable of improving the course of treatment and follow-up of high-risk patients, especially those with incipient caries. About limitations, this study was *in vitro* with limited sample size, and it is better to compare more devices and diagnostic methods with each other.

## Conclusion

With regard to its significantly low percentage of false negative responses and high sensitivity in the diagnosis of dental caries, VistaCam IX Proxi is suitable for caries detection, especially enamel caries, and can be a valuable adjunct to the bitewing radiography technique in the clinical setting. VistaCam IX Proxi is also applicable for preventive measures and follow-up of pediatric patients and individuals for whom radiography is difficult or contraindicated. 
